# Induction of APOBEC3-mediated genomic damage in urothelium implicates BK polyomavirus (BKPyV) as a hit-and-run driver for bladder cancer

**DOI:** 10.1038/s41388-022-02235-8

**Published:** 2022-02-22

**Authors:** Simon C. Baker, Andrew S. Mason, Raphael G. Slip, Katie T. Skinner, Andrew Macdonald, Omar Masood, Reuben S. Harris, Tim R. Fenton, Manikandan Periyasamy, Simak Ali, Jennifer Southgate

**Affiliations:** 1 Jack Birch Unit for Molecular Carcinogenesis, Department of Biology and York Biomedical Research Institute, University of York, Heslington, York YO10 5DD UK; 2 Faculty of Biological Sciences, School of Molecular and Cellular Pathology, University of Leeds, Leeds, UK; 3 Leeds Kidney Unit, St James’s University Hospital, Leeds, UK; 4 College of Biological Sciences, University of Minnesota, Minneapolis, MN 55455 USA; 5 School of Biosciences, University of Kent, Canterbury, CT2 7NJ UK; 6 Department of Surgery & Cancer, Faculty of Medicine, Imperial College London, London, UK; 7 Present Address: School of Cancer Sciences, Cancer Research UK Centre, Faculty of Medicine, University of Southampton, Southampton, SO16 6YD UK; 8 Present Address: Disease Intervention Technology Laboratory (DITL), Institute of Molecular and Cell Biology (IMCB), 8A Biomedical Grove, Neuros/Immunos, #06-04/05, Singapore, 138648 Singapore

**Keywords:** Bladder cancer, Infection, Transcriptomics, Homologous recombination

## Abstract

Limited understanding of bladder cancer aetiopathology hampers progress in reducing incidence. Mutational signatures show the anti-viral apolipoprotein B mRNA editing enzyme catalytic polypeptide (APOBEC) enzymes are responsible for the preponderance of mutations in bladder tumour genomes, but no causative viral agent has been identified. BK polyomavirus (BKPyV) is a common childhood infection that remains latent in the adult kidney, where reactivation leads to viruria. This study provides missing mechanistic evidence linking reactivated BKPyV-infection to bladder cancer risk. We used a mitotically-quiescent, functionally-differentiated model of normal human urothelium to examine BKPyV-infection. BKPyV-infection led to significantly elevated APOBEC3A and APOBEC3B protein, increased deaminase activity and greater numbers of apurinic/apyrimidinic sites in the host urothelial genome. BKPyV Large T antigen (LT-Ag) stimulated re-entry from G0 into the cell cycle through inhibition of retinoblastoma protein and activation of EZH2, E2F1 and FOXM1, with cells arresting in G2. The single-stranded DNA displacement loops formed in urothelial cells during BKPyV-infection interacted with LT-Ag to provide a substrate for APOBEC3-activity. Addition of interferon gamma (IFNγ) to infected urothelium suppressed expression of the viral genome. These results support reactivated BKPyV infections in adults as a risk factor for bladder cancer in immune-insufficient populations.

## Introduction

Urothelial (bladder) cancer has a complex natural history with an indolent, frequently recurrent and unpredictably progressive disease path, which converges with a more aggressive route taken by malignancies that can present at advanced muscle-invasive and even disseminated stages. Although smoking is a well-established risk factor for bladder cancer (BLCA), the mutational signatures of bladder tumours show only a minor proportion of the G > T transversions characteristic of DNA damage caused directly by smoke-derived carcinogens [1, 2]. Studies of bladder tumour genomes have identified mutational signatures associated with the anti-viral apolipoprotein B mRNA editing enzyme catalytic polypeptide (APOBEC) family of cytosine deaminase enzymes in up to 93% of cases [3].

Polyomavirus (PyV) infection could provide an alternative route to initiation that would be of particular relevance to immune-insufficient populations. PyV are ubiquitous childhood infections, with 70–80% seroprevalence by adulthood [4]. They are largely asymptomatic in the immuno-competent, but may remain latent in the adult kidney [5]. BK polyomavirus (BKPyV) resides latent in differentiated renal proximal tubule cells and reactivation at times of immune-insufficiency leads to sloughing of actively infected “decoy” cells (into the urine), to limit kidney damage [6]. PyV DNA can be detected in the urine of immuno-competent individuals with a frequency that increases with age (to ≥ 30% in the over 50 s for BKPyV) [5, 6]. The only published study of BLCA risk (*n* = 3782), found a higher incidence of bladder tumours (15.8%) in the 133 patients with previous urine cytology evidence of BKPyV infection (OR3.4, *p* < 0.001) [7].

**Fig. 1:**
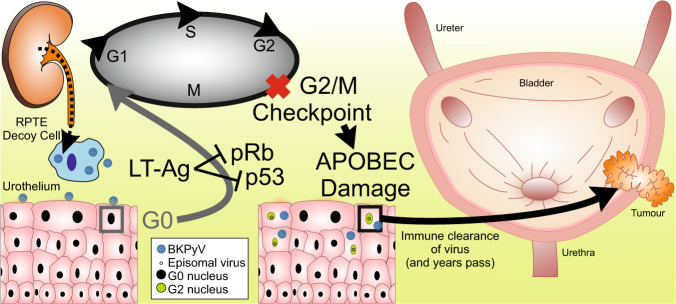
Schematic model of BKPyV hit-and-run carcinogenesis hypothesis. Immune-insufficiency leads to reactivation of latent BKPyV, sloughing of actively-infected renal “decoy” cells and BKPyV viruria. BKPyV infects the G0-arrested urothelium but remains episomal. In infected urothelial cells, BKPyV LT-Ag inhibits host retinoblastoma (pRb) and disables p53, releasing urothelial cells from G0 into the cell cycle for arrest at the G2/M checkpoint. BKPyV stimulates APOBEC3 enzyme activity and causes host genome damage that inactivates tumour suppressors. The immune system clears the virus but initiated cells persist and over a period of years expand to form a tumour.

The large T antigen (LT-Ag) of BKPyV has been shown to induce APOBEC3B expression [8, 9]. However, studies of bladder tumours fail to identify viral DNA or RNA, with fewer than 4% positives reported in the largest study of 689 cases [10]. A successful PyV life-cycle requires the viral genome to remain episomal (*i.e*., non-integrated). The hypothesis of this study is that the episomal life-cycle of BKPyV in the urothelium is capable of initiating bladder tumours via APOBEC-mediated damage of the host genome. This is a so-called “hit-and-run” mode of carcinogenesis whereby the presence of virus causes the initiating inactivation mutation of tumour suppressor genes decades before a tumour forms. Subsequent immune clearance of BKPyV leads to its absence from later stages of tumour development (summarised Fig. 1).

The asymptomatic nature of BKPyV infection means that clinical sampling during reactivated adult BKPyV infection is difficult and since BKPyV is human-specific, in vivo models are not applicable. This study was designed to test the hypothesis that BKPyV-infection of normal human urothelium brings about signature changes that are associated with BLCA and compatible with hit-and-run carcinogenesis. Human urothelium is a low-turnover mitotically-quiescent epithelium where cells typically reside in G0 [11]. To address limitations in previous urothelial studies which have employed actively-dividing, undifferentiated cell cultures [12–14], this study used a mitotically-quiescent (G0-arrested), stratified and differentiated barrier-forming in vitro model of normal human urothelium, with biological replicates to reflect donor diversity [15]. Interferon-γ (IFNγ) has previously been clinically-associated with BKPyV infection [16] and suggested to reduce BKPyV-infection progression in renal cell cultures [17]; therefore we investigated its potential for regulating urothelial anti-viral self-defence mechanisms.

## Results

### Expression of the BKPyV genome during infection of normal human urothelium

**Fig. 2:**
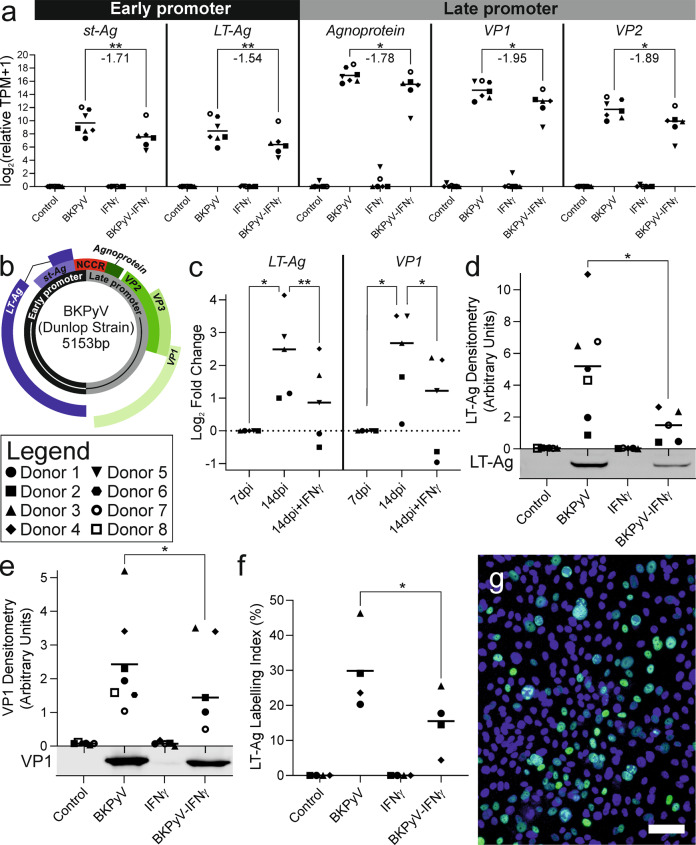
Expression of viral genes and proteins during BKPyV infection of normal human urothelium. **a** mRNAseq analysis of BKPyV gene expression at 14 days post infection (dpi) showed late promoter genes including *Agnoprotein* were the most expressed viral genes, whilst early promoter genes such as *LT-Ag* were less transcriptionally-active. All viral gene expression was significantly suppressed by the indirect actions of IFNγ on the urothelium; however, the efficacy was widely variable between donors. Statistically-significant differences are indicated by stars with the mean log_2_ fold change in gene expression reported beneath (*n* = 6 or 7 independent donors). **b** BKPyV genome map showing the non-coding control region (NCCR) which regulates both the early and late genes that are expressed in opposing orientations. **c** RT-qPCR analysis of BKPyV *LT-Ag* and *VP1* transcript abundance in normal human urothelial (NHU) cell cultures. Data is displayed as log_2_ fold-change normalised to abundance at 7 dpi. *LT-Ag* and *VP1* abundance increased significantly between 7 and 14 dpi. The addition of IFNγ led to a significant reduction in *LT-Ag* and *VP1* abundance at 14 dpi. **d** 14 dpi western blot densitometry for large T antigen (LT-Ag) showing significant reduction by IFNγ (mean log_2_ fold change −1.77), with exemplar blot image from a representative patient shown below the x-axis. Truncated LT-Ag (truncT-Ag) was also expressed; densitometry analysis of truncT-Ag and whole blots for all donor lines probed with LT-Ag can be found in Supplementary Fig. 1 and the β-actin loading controls in Supplementary Fig. 2. **e** 14 dpi western blot densitometry for viral capsid protein 1 (VP1) showing significant (mean log_2_ fold change −0.64) reduction by IFNγ, with exemplar blot image from a representative patient shown below x-axis. Full VP1 blots for all donor lines are shown as Supplementary Fig. 3 and the β-actin loading controls in Supplementary Fig. 2 (*n* = 5 independent donors). **f** LT-Ag indirect immunofluorescence labelling index found a mean of 29.8% of urothelial cells expressed detectable protein. *n* > 2900 cells per condition per donor. **g** Exemplar LT-Ag indirect immunofluorescence image from Donor 3 BKPyV-infected urothelial cells (all conditions shown in Supplementary Fig. 4). Scale bar denotes 100 μm. Significance was assessed in panels **a** by LRT test and **c**–**f** by paired t-test.

In this first transcriptomic study of BKPyV-infection of normal human urothelium, we employed a mitotically-quiescent and functionally-differentiated tissue model [15] that reflects human diversity in cultures from multiple different donors (*n* = 7 for mRNAseq). All genes of the BKPyV genome were expressed at 14 days-post-infection (dpi), with *Agnoprotein* the most (mean relative TPM = 157,998) and *LT-Ag* the least (mean relative TPM = 699) expressed transcripts (Fig. 2a with genome map as Fig. 2b). Overall, the higher expression of *Agnoprotein*, *VP1* and *VP2* genes indicated greatest activity from the late promoter (Fig. 2a, b). In cultures where IFNγ was added for the 7–14 dpi period, expression of all viral genes was significantly reduced by an average log_2_ fold change of −1.78 (Fig. 2a). However, the ability of human urothelium to frustrate BKPyV gene expression in the presence of IFNγ was highly variable between donors (the range of suppression of all viral genes in different donors was from an average log_2_ fold change of −0.60 for Donor 3 to −5.95 for Donor 5; Fig. 2a).

In all infected cultures, *LT-Ag* and *VP1* transcript expression increased from 7 dpi to 14 dpi (Fig. 2c). In cultures from donors 1&2, the addition of IFNγ at 7 dpi reduced the *LT-Ag* and *VP1* transcript burden by 14 dpi, whereas in the remaining three donors tested, the increase was merely attenuated (Fig. 2c).

Western blotting for LT-Ag and VP1 found both were significantly reduced by addition of IFNγ (Fig. 2d, e). Indirect immunofluorescence showed that on average 29.8% of cells had detectable labelling of LT-Ag and the addition of IFNγ led to significantly fewer infected cells being detected in cultures at 14 dpi (Fig. 2f, g).

### Global analysis of the human urothelial transcriptome and cell cycle initiation by BKPyV

**Fig. 3:**
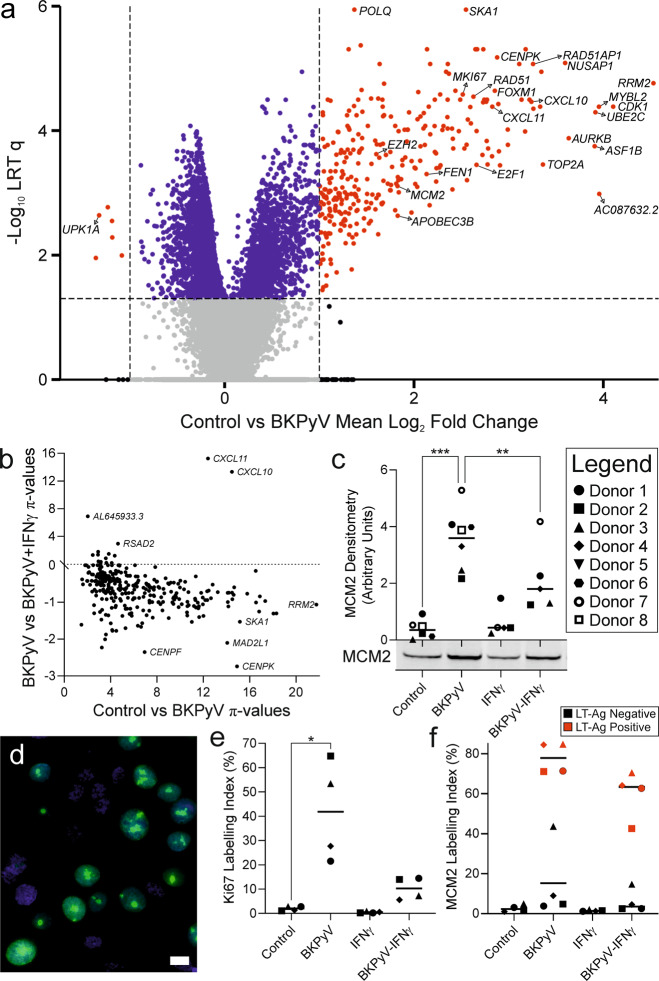
mRNAseq analysis of differentiated human urothelium post BKPyV-infection. **a** Volcano plot highlighting the significant induction of cell cycle and DNA-damage genes 14 dpi with BKPyV compared with controls (*n* = 7 independent donors). **b** The 305 genes significantly induced by BKPyV-infection are plotted to show their induction (x-axis) against the effect of post-infection addition of IFNγ (y-axis). Addition of IFNγ suppressed expression of the vast majority of BKPyV-induced genes (*t* = −17.57; *p* = 1.97 × 10^−47^). The chemokines *CXCL10* and *CXCL11* were notable exceptions, where the addition of IFNγ further increased expression. **c** Western blot densitometry for the DNA replication licensing factor “MCM2” showing significant mean 31-fold induction (*p* < 0.001) at 14 dpi with BKPyV and significant inhibition by IFNγ (*p* < 0.01); a representative blot image from a single patient shown is shown below the x-axis. Whole MCM2 blots for all donors are shown in Supplementary Fig. 6 and the β-actin loading controls in Supplementary Fig. 2. **d** Indirect immunofluorescence labelling of Ki67 (green) in BKPyV-infected urothelial tissues shows nearly all Ki67-positive cells have few large nucleolar granules, characteristic of the G2 cell cycle stage. A single image of BKPyV-infected cells is shown here and larger images of all conditions in a representative donor are shown in Supplementary Fig. 7. DNA was stained with Hoechst 33258 (blue). White scale bar denotes 10 μm. **e** Ki67 labelling indices for quiescent, G0-arrested control cultures were low (mean = 1.99% ± 0.94). BKPyV infection led to a significant mean 27-fold (*p* = 0.0319) increase in Ki67 labelling index (mean = 41.8% ± 20.62). **f** BKPyV infection also led to a significant increase in MCM2 labelling index (*p* = 0.0281; Supplementary Fig. 9). When cells from infected cultures were separated into LT-Ag positive and negative populations, there was a mean 5.6-fold ( ± 4.0) increase in MCM2 labelling of LT-Ag negative cells from BKPyV-infected cultures, implying a field effect. The red colouration for LT-Ag positive cells applies only to panel **f**.

Analysis of the human urothelial transcriptome at 14 dpi with BKPyV by mRNAseq found there was a significant (*q* < 0.05) >2-fold induction of 305 transcripts (Fig. 3a). The major task of any PyV infection of the urinary tract is the initiation of proliferation, since the target epithelia (namely proximal tubule and urothelium) are not actively dividing tissues and both are predominantly (>99%) out-of-cycle, arrested in G0. Gene set enrichment analysis (GSEA; Supplementary Table 1) suggested mitotically-quiescent urothelial cells, once infected, initiated transcriptional programmes related to cell cycle re-entry (specifically the G2/M checkpoint) and DNA damage repair (Supplementary Fig. 5a, b). A small number of genes were significantly reduced; these included the uroplakin genes *UPK1A*, *UPK2* and *UPK3A*, suggesting superficial urothelial differentiation was affected by BKPyV infection (Fig. 3a). The addition of IFNγ to BKPyV-infected urothelium (at 7 dpi until 14 dpi) significantly suppressed the majority of the 305 BKPyV infection-induced genes (*t* = −17.57; *p* = 1.97 × 10^−47^) but further augmented expression of *CXCL10* and *CXCL11* (Fig. 3b). CXCL10 and CXCL11 are chemokine ligands that recruit the leukocytes which drive the IFNγ-response, including Th1 polarisation, leukocyte activation and suppression of infection-induced genes in the urothelium (reviewed [18, 19]).

Urothelial cell re-entry into the cell cycle was supported by significant (*q* < 0.001) induction of *MCM2* and *MKI67* transcript induction (mean log_2_ fold change = 5.69 and 2.11, respectively; Supplementary Fig. 5c, d). IFNγ exposure reduced mean *MCM2* and *MKI67* transcript expression in BKPyV-infected cultures compared with infection alone but, due to the variance in the IFNγ-response, these changes were not statistically significant (Supplementary Fig. 5c, d). At the protein level MCM2 was significantly induced by BKPyV-infection (*p* < 0.001) and that increase was significantly suppressed by IFNγ (*p* < 0.01; Fig. 3c & Supplementary Fig. 6). Cell cycle stage analysis of Ki67 immunolocalisation [20] showed both a significant (*p* = 0.03) increase in the proportion of positive cells within BKPyV-infected cultures and indicated that Ki67-positive cells were overwhelmingly in the G2 stage of the cell cycle, as identified by the few large nucleolar granules observed in each nucleus (Fig. 3d, e, respectively; Supplementary Fig. 7). G2-arrest in BKPyV-infected cultures was supported transcriptomically by significant enrichment of gene sets associated with negative regulation of the G2 to M transition and experimentally-induced G2-arrest (Supplementary Fig. 5e, f). Furthermore, analysis of nuclei in urothelial cells from BKPyV-infected cultures indicated a significant increase in nuclear size for LT-Ag labelled cells, supporting cell cycle progression beyond S-phase (Supplementary Fig. 8). Indirect immunofluorescence co-labelling of MCM2 and the LT-Ag revealed that infection with BKPyV triggered a significant increase in the proportion of MCM2 positive cells and that significantly fewer cells became MCM2 positive in the presence of IFNγ (Supplementary Fig. 9). Using LT-Ag labelling to differentiate positive from negative cells in infected cultures indicated that MCM2 was also elevated 5.6-fold in LT-Ag negative cells within BKPyV-exposed cultures (Fig. 3f).

### Cell cycle entry is driven by LT-Ag inhibition of Retinoblastoma protein

**Fig. 4:**
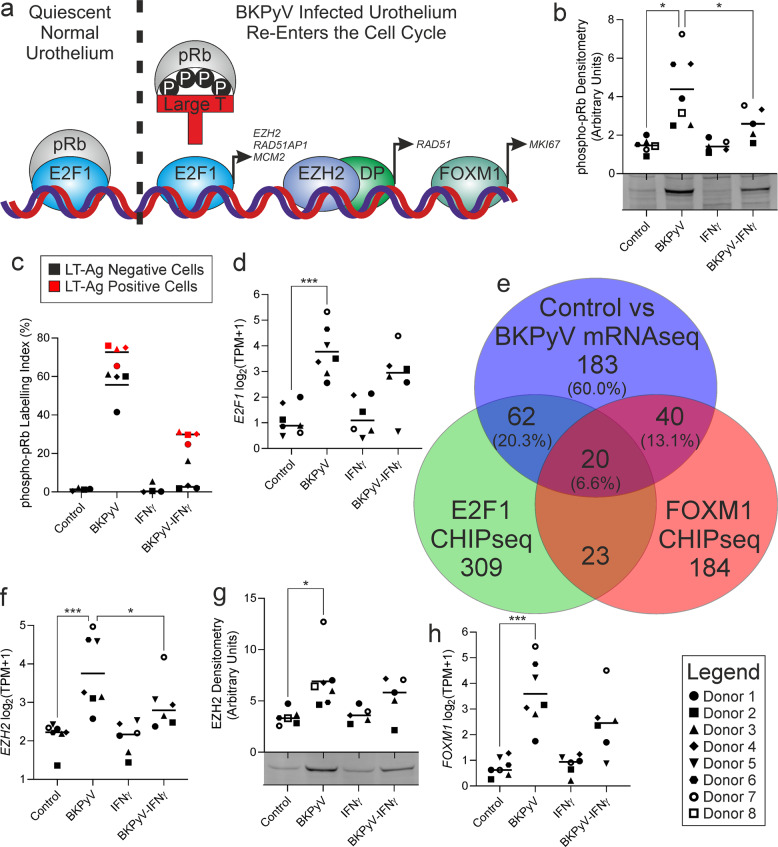
BKPyV stimulates urothelial cell cycle re-entry by inactivating phosphorylation of retinoblastoma protein. **a** Schematic summary of proposed BKPyV cell cycle regulation. pRb Retinoblastoma protein, DP Dimerization partners, P Phosphorylation. **b** The RB1 transcript that encodes pRB showed no changes in response to infection or IFNγ (Supplementary Fig. 11a); however, western blotting of pRb phosphorylated at serine 807/811 showed a significant infection-associated increase (mean 3.0-fold) that was suppressed by IFNγ. A representative blot from a single donor is shown here, with full blots for all donors in Supplementary Fig. 11b and the β-actin loading controls in Supplementary Fig. 2. **c** BKPyV infection also led to a significant mean 88-fold increase in phosphorylated-pRb labelling index (*p* < 0.01; representative images are shown in Supplementary Fig. 12). When cells from infected cultures were split into LT-Ag positive and negative populations, there was a mean 63.1-fold ( ± 57.1) increase in phosphorylated-pRb labelling of LT-Ag negative cells from BKPyV-infected cultures from all donors compared to non-infected controls, supporting the proposed field effect. The red colouration for LT-Ag positive cells applies only to panel **c**. **d** mRNAseq showed significant induction of the pRb-regulated E2F1 transcription factor, which was induced more prominently than other members of the E2F family (Supplementary Fig. 13). **e** Comparison of genes significantly 2-fold induced by BKPyV-infection with those reported to possess proximal E2F1 [21] and FOXM1 [23] ChIP-seq peaks, suggested 40% of the BKyV-induced transcriptome may be activated by these transcription factors. E2F1 ChIPseq peaks [21] and BKPyV-induced genes had a significant overlap of 82 genes (exact hypergeometric probability *p* < 4.95 × 10^−106^; representation factor = 38.6). There was a 60-gene overlap (exact hypergeometric probability *p* < 1.30 × 10^−80^; representation factor = 43.8) between genes up-regulated upon BKPyV-infection and those associated with FOXM1 binding [23]. Gene lists provided as Supplementary Fig. 14. Increased E2F and FOXM1-activity was further supported by GSEA (Supplementary Fig. 10d–f and 10h). **f** and **g** mRNAseq and western blotting respectively show a BKPyV-mediated increase in expression of the DREAM complex member EZH2. EZH2 joins the polycomb repressive complex 2 (PRC2) dimerization partners to drive transcription as shown by GSEA (Supplementary Fig. 10g). A representative blot from a single donor is shown here, with full EZH2 blots shown in Supplementary Fig. 15 and the β-actin loading controls in Supplementary Fig. 2. **h** mRNAseq shows significant induction of *FOXM1* transcription by BKPyV-infection.

BKPyV regulates the cell cycle in part via LT-Ag interactions through its LxCxE domain with Retinoblastoma protein (pRb; Fig. 4a). GSEA identified pRb disruption (Supplementary Fig. 10a–c), and whilst *RB1* transcription was unchanged, phosphorylation of pRb was significantly (*p* < 0.05) both increased by BKPyV and reduced by IFNγ exposure (western blotting in Fig. 4b; Supplementary Fig. 11). Indirect immunofluorescence co-labelling of phosphorylated-pRb and the LT-Ag revealed BKPyV triggered an increase in phosphorylated-pRb labelled cells and that significantly fewer cells became positive in the presence of IFNγ (both *p* < 0.01; Supplementary Fig. 12). Using LT-Ag co-labelling (to distinguish infected from non-infected cells in cultures), 63.1-fold more cells were positive for phosphorylated-pRb in the LT-Ag negative fraction of BKPyV-exposed cultures compared to control cultures (Fig. 4c). pRb phosphorylation and cell cycle re-entry by infected and adjacent cells could contribute to tumour growth (promotion) of initiated cells.

Expression of the *E2F1* gene was increased by BKPyV-infection (*p* < 0.001; Fig. 4d) along with other members of the E2F family (Supplementary Fig. 13). The inhibition of pRb either by LT-Ag-binding or phosphorylation increased E2F-activity, as evidenced by gene-set enrichment of E2F1 targets in BKPyV infected cells (Supplementary Fig. 10d–f). To confirm this finding we analysed the overlap in genes associated with previously reported E2F1 ChIPseq peaks [21] and BKPyV-induced genes and found a significant (exact hypergeometric probability *p* < 4.95 × 10^−106^; representation factor = 38.6) overlap of 82 genes (Fig. 4e; gene lists in Supplementary Fig. 14). One of the genes induced by E2F1 was the histone methyltransferase *EZH2* (Fig. 4f, g), which joins the polycomb repressive complex 2 (PRC2) dimerization partners to drive transcription [22]. Involvement of EZH2/PRC2 was supported by GSEA (Supplementary Fig. 10g). Transcription and protein expression of EZH2 was induced by BKPyV infection and reduced by IFNγ (Fig. 4f–g; Supplementary Fig. 15).

Expression of the late cell cycle-associated transcription factor *FOXM1* was also induced by BKPyV infection (Fig. 4h) and GSEA supported FOXM1-activity during infection (Supplementary Fig. 10h). There was a substantial (exact hypergeometric probability *p* < 1.30 × 10^−80^; representation factor = 43.8) overlap between genes up-regulated upon BKPyV infection and those associated with FOXM1 binding [23], indicative of these genes being transactivated by FOXM1 (Fig. 4e; gene lists in Supplementary Fig. 14). Taken together, increased expression and activities of E2F1 and FOXM1 accounted for 40% of the transcriptome induced by BKPyV-infection (Fig. 4e).

### Homologous recombination and p53 stabilisation during BKPyV-infection

**Fig. 5:**
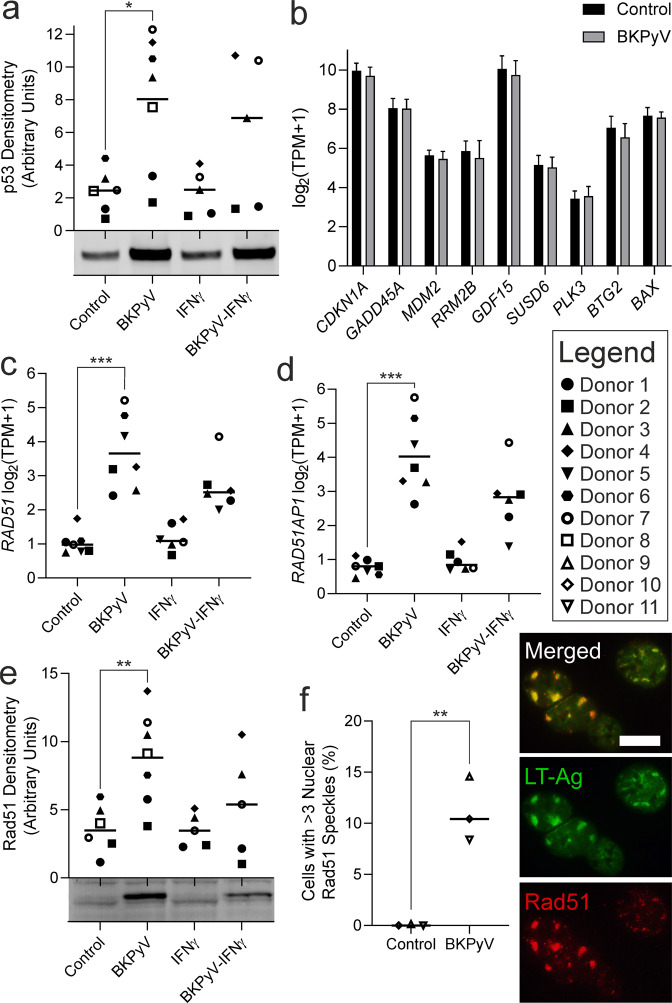
Homologous recombination was induced by BKPyV-infection of human urothelium. **a** Western blotting for p53 showed significant protein stabilisation (no increase in transcription was observed) during BKPyV infection. Stabilisation of p53 in the nucleus was confirmed by indirect immunofluorescence. A representative blot from a single donor is shown here, with full p53 blots shown in Supplementary Fig. 16 and the β-actin loading controls in Supplementary Fig. 2. **b** There was no increased expression of verified p53 target genes [51]. **c** *RAD51* and **d** *RAD51AP1* transcripts were significantly induced by BKPyV-infection. **e** Western blotting confirmed significant Rad51 protein induction (mean 2.7-fold) and phosphorylation was observed as a slight increase in molecular weight when Rad51 was induced by BKPyV. A slight increase in Rad51 molecular size during infection indicated possible activating-phosphorylation of the induced Rad51 by the Chk1 kinase [24], whose transcription was also significantly induced. A representative blot from a single donor is shown here, with full Rad51 blots shown in Supplementary Fig. 17 and the β-actin loading controls in Supplementary Fig. 2. **f** Analysis of indirect immunofluorescence for Rad51 found nuclear speckles were significantly increased in BKPyV infection (from mean 0.05% positive cells in controls to 11.13% in BKPyV-infected; images in Supplementary Fig. 17). In some cells, indirect immunofluorescence revealed LT-Ag and Rad51 protein co-localisation to large granular deposits in the nuclei of BKPyV-infected urothelial cells. Scale bar denotes 10 μm, immunofluorescence performed on *n* = 3 independent donors with representative images shown.

GSEA revealed a DNA-damage response to BKPyV-infection and specifically, genes implicated in homologous recombination and displacement loop structures (Supplementary Figs. 5b and 10i, j). Stabilisation of p53 protein was observed by western blotting and indirect immunofluorescence during BKPyV infection (Fig. 5a; Supplementary Fig. 16); however, this stabilisation was not associated with increased transcription of p53 target genes consistent with LT-Ag inhibition (Fig. 5b). *RAD51* and *RAD51AP1* were significantly induced by BKPyV (mean log_2_ fold change 3.50 and 4.47, respectively; both *p* < 0.001; Fig. 5c, d). Rad51 and Rad51AP1 play roles in the formation of single-stranded DNA displacement loops, which are candidate substrates for cytosine deamination by APOBEC3 proteins. Increased Rad51 protein was confirmed by western blotting, where a slight increase in molecular size indicated possible activating-phosphorylation of the induced Rad51 by the Chk1 kinase [24], whose transcription was also significantly induced (Fig. 5e; Supplementary Fig. 17). Indirect immunofluorescence labelling of Rad51 identified nuclear speckles formed during BKPyV infection (Supplementary Fig. 17) and image analysis showed the appearance of nuclear speckles was significant (*p* = 0.0037; Fig. 5f). The LT-Ag of JCPyV was previously shown to activate the *RAD51* promoter and the two proteins were shown to co-localise in JCPyV infections [25]. Labelling of Rad51 identified nuclear speckles forming during BKPyV-infection and co-localisation of Rad51 with the BKPyV LT-Ag (Fig. 5f).

### Induction of APOBEC-activity and DNA damage by BKPyV-infection

**Fig. 6:**
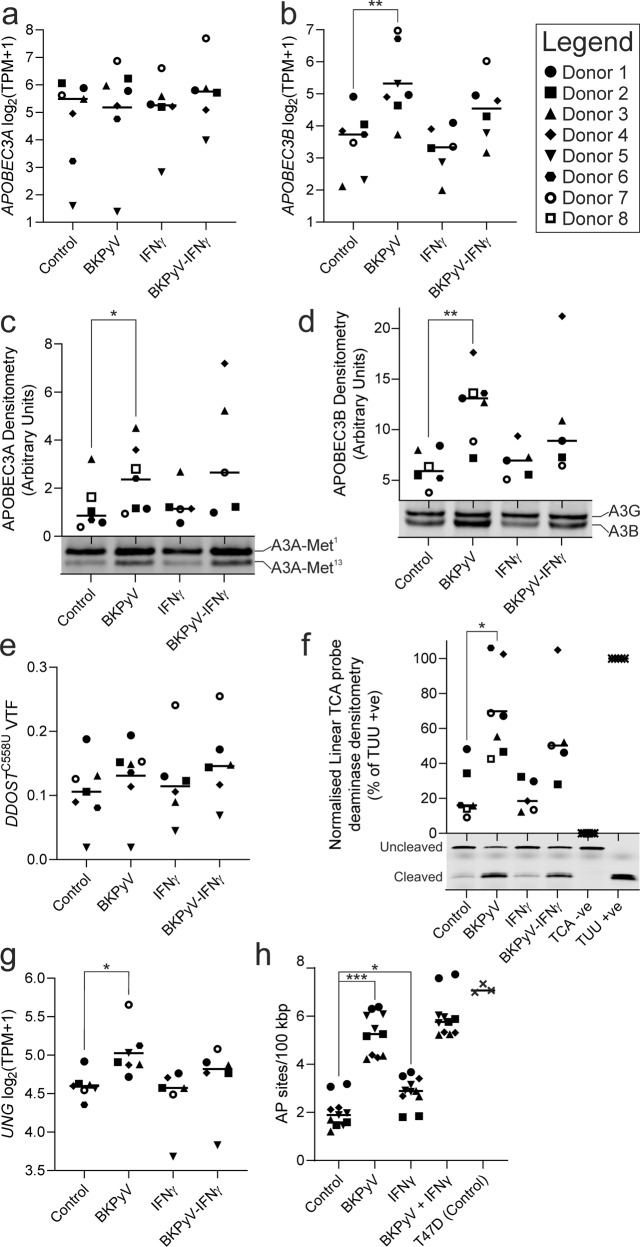
APOBEC3 expression and activity in normal human urothelium. mRNAseq analysis of *APOBEC3A* and *APOBEC3B* transcripts found only *APOBEC3B* was significantly (mean log2 fold change 1.78 ± 1.31; *q* = 0.0023) induced by BKPyV-infection (**a** and **b**, respectively). mRNAseq expression data for the whole APOBEC enzyme family is provided as Supplementary Fig. 18. **c** Western blotting for APOBEC3A found a significant (*p* = 0.0133) 2.04-fold induction by BKPyV infection (whole blots for all donors shown in Supplementary Fig. 20); note “A3A”; full-length (“Met^1^”) and truncated protein from the internal Met^13^ start site were analysed together as both possess catalytic activity [29]. **d** APOBEC3B (“A3B”) and APOBEC3G (“A3G”) bands appear close together on the blots and densitometry shown here is for APOBEC3B only (as APOBEC3G expression was not altered; Supplementary Fig. 20). APOBEC3B protein was significantly (mean 1.93-fold) induced by BKPyV infection (*p* = 0.0025). Western blot densitometry and TPM for APOBEC3B were significantly correlated (Pearson Rho = 0.77; *p* = 0.0001). Densitometry analysis suggested that during BKPyV infection, the ratio of APOBEC3A to APOBEC3B protein was 1.95:1 ( ± 1.18; *n* = 7). **c** and **d** Representative APOBEC blot images from a single donor are shown here, with full blots for all donors shown in Supplementary Fig. 20 and the β-actin loading controls in Supplementary Fig. 2. **e** Variant transcript frequency (VTF) for APOBEC-mediated C > U editing of *DDOST* RNA at cytosine 558 quantified by mRNAseq was increased slightly, but not significantly, in 6/7 donors by BKPyV infection. Donors 5 and 7 appeared to show an IFNγ-mediated induction of APOBEC3A-function. Across all mRNAseq samples (*n* = 26) *DDOST* C558U variant transcript frequency (VTF) was significantly correlated with *APOBEC3A* TPM (Pearson Rho = 0.867; *p* < 0.0001) but not *APOBEC3B* TPM (Pearson Rho = 0.368; *p* = 0.0642; Supplementary Fig. 21). **f** Deaminase assays for urothelial cell lysates against a linear RTCA probe, preferentially targeted by APOBEC3B, showed significant induction of activity following BKPyV infection (mean log_2_ fold change 1.66 ± 1.05; *p* = 0.0313; gels for all donors shown in Supplementary Fig. 22). Deaminase assays were also performed against a YTCA hairpin probe, in the presence and absence of RNA, to evaluate APOBEC3A activity (Supplementary Fig. 24). **g** mRNAseq expression data for the uracil-DNA glycosylase gene *UNG* found it was significantly induced by BKPyV-infection (*q* = 0.0213). **h** An apurinic/apyrimidinic (AP) sites assay was performed on genomic DNA from five independent donors analysing 2–3 independent cultures per donor. T74D breast cancer cells were included as a positive control known to generate apurinic/apyrimidinic sites in their genomes [50]. The legend in the top right identifies the donor by specific point shapes in all dot plot panels.

To explore the possible involvement of APOBEC3 cytidine deaminases (enzyme family expression data Supplementary Fig. 18), we investigated the expression of *APOBEC3A* and *APOBEC3B*, which have been implicated in the increased mutational burden in virally-driven cancers, such as HPV [26]. *APOBEC3A and APOBEC3B* transcription was highly variable between donors (Fig. 6a, b). *APOBEC3A* transcript was increased by BKPyV-infection in some donors (Donors 6 & 7 showed > 2-fold induction), but this trend was not replicated in all (Fig. 6a). *APOBEC3B* transcript was significantly induced > 2-fold in 5 of 7 BKPyV-infected donors (mean log2 fold change 1.78 ± 1.31; *p* < 0.01; Fig. 6b). The *APOBEC3B* promoter was recently shown to be a direct target of pRb/E2F signalling [27]; which is consistent with the pRb/E2F and APOBEC3B induction reported here. When *APOBEC3B* expression was compared with viral transcripts, its TPM value was most significantly correlated with *LT-Ag* (Pearson Rho = 0.98, *p* = 1.407 × 10^−9^; Supplementary Fig. 19). However, the *APOBEC3A* transcript abundance was not correlated with BKPyV transcript expression (Supplementary Fig. 19). *APOBEC3A* and *APOBEC3B* expression can be induced by interferons, but this has been reported to be limited in urothelial cancer cell lines as compared to breast cancer lines [28]. Interestingly, IFNγ did not stimulate expression of APOBEC3A/APOBEC3B in NHU cells (Fig. 6a, d), nor was APOBEC3A/APOBEC3B expression increased by IFNγ in BKPyV-infected NHU cells (Fig. 6a, d).

APOBEC3 protein abundance was studied by western blotting (Fig. 6c, d; Supplementary Fig. 20). APOBEC3A has two enzymatically-active isoforms, with a smaller variant generated by internal translation initiation at a methionine at position 13 (Met^13^) [29]. Both APOBEC3A isoforms were increased in all donors following BKPyV infection (*p* = 0.0025; Fig. 6c), although there was large variation in the magnitude of increase. In cells from Donors 4 & 5, APOBEC3A protein abundance in BKPyV-infected cultures was increased >2-fold by IFNγ exposure (Fig. 6c). APOBEC3B was significantly increased by BKPyV infection (*p* = 0.0054; Fig. 6d). Western blot densitometry was significantly correlated with TPM for both APOBEC3A and APOBEC3B (*p* = 0.0147 and *p* = 0.0003, respectively). Densitometry analysis suggested that during BKPyV infection, the ratio of APOBEC3A to APOBEC3B protein was 1.95:1 ( ± 1.18; *n* = 7).

A recent study identified RNA-editing of the *DDOST* transcript at cytosine 558 to be a common activity of APOBEC3A that was not observed with APOBEC3B [30]. *DDOST* transcript editing was mildly increased in 6/7 donors by BKPyV infection (25.2% increase ± 23.9; Fig. 6e). Across all mRNAseq samples (n = 26) *DDOST* C558U variant transcript frequency (VTF) was significantly correlated with *APOBEC3A* TPM (Pearson Rho = 0.803; *p* = 5.205 × 10^−7^) but not *APOBEC3B* TPM (Pearson Rho = 0.278; *p* = 0.167; Supplementary Fig. 21).

APOBEC3-activity was assessed by deaminase assays using single-stranded DNA probes conjugated to fluorochromes (Fig. 6f). BKPyV-infection significantly increased deaminase-activity against a linear probe with an RTCA motif that is the substrate preference of APOBEC3B (mean log_2_ fold change 1.66 ± 1.05; *p* = 0.0303; Fig. 6f; Supplementary Fig. 22). Linear regression analysis confirmed significant relationships between deaminase activity and APOBEC3B protein abundance (*F* = 33.19; df = 21; *p* < 0.0001), and to a lesser extent APOBEC3A protein (*F* = 5.030; df = 21; *p* = 0.0358; Supplementary Fig. 23). Further studies using a hairpin YTCA probe, designed to favour APOBEC3A-deamination [31], also showed increased activity following BKPyV-infection. The addition of RNA, demonstrated to inhibit APOBEC3B, but not APOBEC3A activity [31], had no effect on deamination of the hairpin substrate, suggesting that APOBEC3A-activity was induced by BKPyV infection (Supplementary Fig. 24).

APOBEC-activity in the genome converts cytosine to uracil, which requires excision by the uracil DNA glycosylase enzyme to leave an apurinic/apyrimidinic (AP) site suitable for subsequent repair. The uracil-DNA glycosylase enzyme gene “*UNG*” was significantly induced by BKPyV-infection (*q* = 0.0213; Fig. 6g). Furthermore, the increased deaminase activity observed in BKPyV infection (Fig. 6f) was associated with significant (*p* < 0.001) damage to the host genome, measured as an increase in AP sites (Fig. 6h). Linear regression analysis confirmed a significant relationship between AP sites and APOBEC3B protein abundance (*F* = 9.033; df = 17; *p* = 0.008) but not APOBEC3A (*F* = 1.190; df = 17; *p* = 0.185; Supplementary Fig. 25).

### Association of LT-Ag with Rad51 and APOBEC enzymes within the nucleus of infected cells

**Fig. 7:**
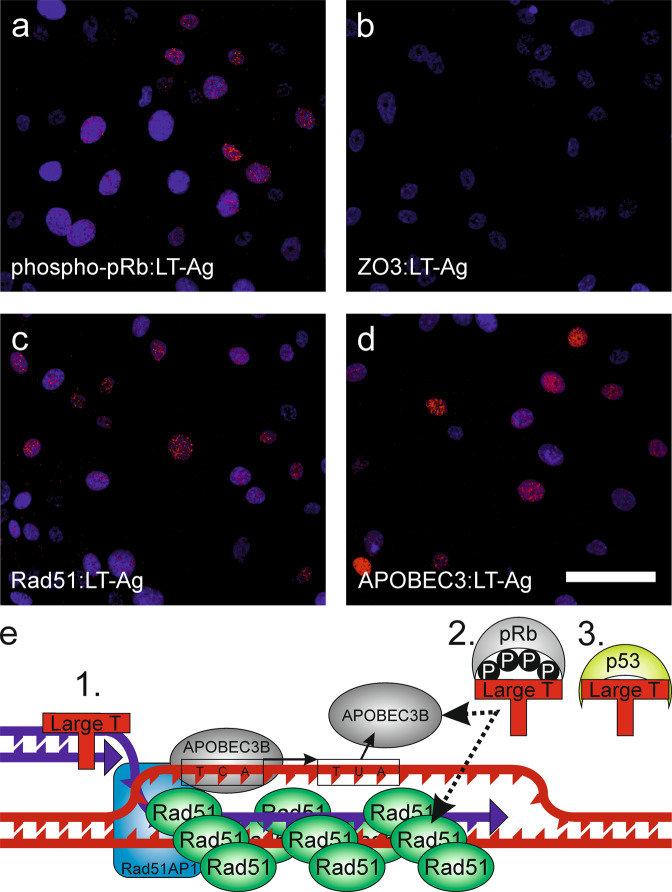
Proximity ligation assays of human urothelium during BKPyV-infection. Proximity ligation assays indicated that nuclear complexes formed involving large T antigen (LT-Ag) and **a** phospho-Retinoblastoma (phospho-pRb), **b** Zonula occludins 3 (ZO3) is an intercellular tight junction protein and included here as a non-nuclear-localized negative control, **c** Rad51 and **d** APOBEC3 proteins. Scale bar denotes 50 μm, images from one representative donor during BKPyV-infection are shown here with images from three donors, including non-infected controls, provided as Supplementary Figs. 26–28. **e** Schematic of hypothetical model of urothelial DNA-damage at displacement loops at the G2/M checkpoint during BKPyV infection. LT-Ag interactions might derive from (1.) its DNA-binding helicase activity or (2.) LT-Ag protein dimerization with another involved protein, such as pRb. (3.) LT-Ag inhibition of p53 is likely important for this process as p53 would normally prevent Rad51 oligomerisation by direct binding [52]. The evidence from this study is most robust for BKPyV-induced APOBEC3B-activity but APOBEC3A protein was consistently more abundant during infection and its role requires further validation.

To understand how APOBEC3-activity relates to viral infection, proximity ligation assays were used to study protein:protein interactions. LT-Ag is a critical biological effector of the BKPyV life-cycle and has previously been shown to interact directly with pRb [32]. Here we used the pRb:LT-Ag interaction as a positive control for proximity ligation assays (Fig. 7a) and included ZO3:LT-Ag as a negative control (zonula occludins 3 (ZO3) is a differentiated urothelial tight junction member and not known to enter the nucleus; Fig. 7b). LT-Ag appeared to co-localise with Rad51 by double-immunolabelling (Fig. 5f) and proximity ligation assays confirmed that the two proteins were frequently localised ≤ 40 nm from one another (Fig. 7c). In addition, APOBEC3 enzymes co-localised with LT-Ag in the nuclei of infected urothelial cells (Fig. 7d). LT-Ag has been shown to bind dsDNA and act as a helicase; however, the precise nature of LT-Ag’s binding partners and complex membership remains to be resolved. We hypothesise that LT-Ag acts as a lynchpin bringing multiple factors together around displacement loops to harness DNA repair enzymes for viral genome replication, resulting in collateral damage to the host genome (summarised Fig. 7e).

## Discussion

Tracking the molecular events leading to epithelial malignancies can be challenging when the carcinogenic event may have occurred decades in the past. Genomic mutational signatures offer insight but, in the case of BLCA, controversy exists between the putative epidemiological risk from smoking and mutational signatures found in tumours [2, 3], which suggests a missing viral agency. Using a tissue-mimetic in vitro model that replicates both the barrier function and mitotic-quiescence of human urothelium in situ, we provide the first experimental evidence that BKPyV can directly infect differentiated human urothelium, driving the host cells into G2, to facilitate the viral life cycle. This is achieved by viral LT-Ag mediated inactivation of the host tumour suppression machinery, including p53 and pRb. A key corollary of these events is the acquisition of host genomic damage through APOBEC3A/B-activity, which we associate with DNA displacement loops formed during homologous recombination at the G2/M checkpoint (summarised Fig. 7e). This evidence supports BKPyV as an infectious agent with the capacity to damage the urothelial genome and initiate carcinogenesis. Urothelial expression of CXCL9/10 in response to BKPyV-infection will recruit host immune cells into the tissue, which release IFNγ to start a cascading reaction that limits viral infection.

BKPyV-infections and BLCA are more common in immunosuppressed patients following solid organ transplant (standardised incidence ratio 1.52 [33]) and in particular kidney transplant (meta-analysis standardised incidence ratio 2.46 [34]). The mean time from renal transplant to BLCA diagnosis was reported as 9.6 years [35], comparable with the disease course in HPV-driven tumours. Other biological processes leading to immune-insufficiency, including ageing, may also be sufficient to trigger BKPyV-reactivation. Renal BKPyV-reactivation can lead to transient reinfection of the urothelium, driving host cell-cycle activity and the APOBEC-mediated genomic damage found in clonal patches of the normal tissue that were recently described [36]. In light of this, we propose due consideration be given to BKPyV vaccine development [37] and subsequent trials with an initial focus on renal transplant patients as an at-risk population where significant benefits in terms of graft outcomes and urinary tract pathologies (including reduced cancer risk) are likely. Viewed through the prism of BKPyV as a risk factor for BLCA, a number of features of the disease that are not currently understood might start to be explained (such as the role for smoking in infection-susceptibility [38] or the pronounced sex bias in BLCA deriving from different infection-responses [39]).

Our work adds to epidemiological studies by providing experimental evidence in differentiated human urothelium that BKPyV-infection could play a causal role in carcinogenesis. Further evidence is required to show that initiating mutations in tumours are BKPyV-infection derived and not the result of other APOBEC-inducing processes. New studies, aimed at mapping the progression of AP sites into mutational signatures and at understanding the selection pressures in post-initiated normal urothelium that promote tumorigenesis, are urgently required.

## Conclusions

This study provides mechanistic evidence that BKPyV can infect and leave APOBEC-induced damage in the urothelial genome. The results support reactivated BKPyV infections as a risk factor for BLCA in immune-insufficient adult populations, including transplant patients and the elderly. This work provides a case for trialling BKPyV vaccination in recipients awaiting renal transplantation.

## Materials and methods

### Normal human urothelial (NHU) cell culture

Eleven independent NHU cell lines of finite (non-immortalised) lifespan were used in this study. The cell lines were established as described [40] using anonymous discarded tissue from renal transplant surgery, with NHS Research Ethics Committee approval. NHU cells were propagated in Keratinocyte Serum-Free Medium (KSFM; 0.09 mM Ca^2+^) supplemented with bovine pituitary extract, recombinant human EGF and 30 ng/ml cholera toxin. Following expansion, NHU cells were differentiated in medium supplemented with adult bovine serum and [Ca^2+^] elevated to 2 mM, according to published methods [15].

### BKPyV infection and IFNγ treatment

BKPyV Dunlop Strain was expanded for use in renal proximal tubule epithelial cell cultures which were scrape harvested at 14 days post-infection, sonicated, and frozen as aliquots at −80 °C. The multiplicity of infection (MOI) was calculated by fluorescent focus unit assay using IncuCyte ZOOM analysis (Essen BioScience, Ann Arbor, MI, USA), as previously reported [41]. All cell cultures in this study were infected with 0.45 μm filtered BKPyV containing medium at MOI = 1 for 3–4 h at 37 °C before the virus-containing medium was removed and cultures continued.

Initial studies found BKPyV-infection of mitotically-quiescent urothelium to be more efficient in differentiated than undifferentiated cultures (Supplementary Fig. 29). For this study, BKPyV infection was evaluated during the pre-lytic phase of infection in differentiated normal human urothelial (NHU) cell cultures which showed no morphological or transcriptomic signs of apoptosis during the 14 day infection period (Supplementary Fig. 30). At 7 days post-infection (dpi) some cultures were exposed to IFNγ (200 U/mL, BioTechne #285-IF) for a further seven days to mimic an immune response to the infection.

### mRNA analysis

Total RNA was collected in TRIzol reagent (Invitrogen).

Selected transcripts were assessed by Reverse Transcribed - quantitative Polymerase Chain Reaction (RT-qPCR) using primers described in Supplementary Methods, with amplification monitored by SYBR Green dye on a QuantStudio™ 3 Real-Time PCR System machine (ThermoFisher) using the ΔΔct method relative to *GAPDH* expression.

Samples from 14 dpi were selected for mRNA sequencing (mRNAseq) using the Illumina NovaSeq 6000 generating 150 bp paired-end reads (Novogene UK, Cambridge, UK). All mRNAseq data has been deposited at GSE174244. Following standard quality control, gene-level expression values in transcripts per million (TPM) were derived against the Gencode v35 human transcriptome using kallisto v0.46.1 [42]. For analysis of the BKPyV transcriptome (Fig. 2a), sequences (derived from BKPyV reference; GenBank NC_001538.1) were appended to the human transcriptome to generate “relative TPMs” as a measure of viral transcript abundance. Reads were also aligned to the human (GRCh38) and BKPyV reference genome assemblies with HISAT2 v2.2.0 [43], and single nucleotide RNA variants in *DDOST* were detected following best practices using GATK v4.1.0 [44], PicardTools v2.20.0 [45], SAMtools v1.10 [46] and VCFtools v0.1.15 [47].

Differentially-expressed genes were identified using the Sleuth v0.30.0 [48] implementation of the likelihood ratio test (LRT), accounting for matched genetic backgrounds, generating Benjamini-Hochberg corrected *q*-values. For volcano plots (performed in R v4.0.4 EnhancedVolcano v1.8.0), fold change values used a TPM + 1 transformation to reduce the influence of low abundance transcripts.

To compare manipulations of the culture model, π-values were calculated as described elsewhere [49]:

$$\pi = log_2\,fold\,change\left( {TPM + 1} \right) \times - log_{10}\,LRT\,q$$

Gene set enrichment analysis (GSEA) was performed using π-values (derived from the Sleuth LRT *q*-values) and the pre-ranked list feature implemented in the python package GSEApy (0.10.2; available at: https://github.com/zqfang/GSEApy). The ranked list of genes was run against the four following Molecular Signatures Database (MSigDB) collections: hallmarks (h.all.v.7.2.symbols.gmt), curated (c2.all.v.7.2.symbols.gmt), gene ontology (c5.all.v.7.2.symbols.gmt), and oncogenic signatures (c6.all.v.7.2.symbols.gmt) (available at: https://data.broadinstitute.org/gsea-msigdb/msigdb/release/7.2/).”

Overlap between genes significantly 2-fold induced by BKPyV-infection and previously reported E2F1/FOXM1 ChIPseq peaks [21, 23] was assessed by calculating the exact hypergeometric probability.

### Indirect immunofluorescence

NHU cell cultures on glass 12-well slides were fixed in methanol:acetone (1:1) for 30 s, air-dried, and stored frozen.

Primary antibodies (detail provided in Supplementary Methods) were applied overnight at 4 °C. Unbound primary antibodies were removed by washing in phosphate-buffer saline (PBS) and secondary antibodies (Goat-anti-Mouse Ig Alexa-488 and Goat-anti-Rabbit Ig Alexa-594, Molecular Probes) were applied for 1 h at ambient temperature. Slides were washed in PBS, with 0.1 µg/ml Hoechst 33258 added to the penultimate wash, before mounting in ProLong Gold Antifade Mountant (ThermoFisher) and visualisation by epifluorescence on a BX60 microscope (Olympus).

Image analysis was performed in ImageJ (v1.53c Java 1.8.0_172) by creating regions of interest (ROI) around the nuclei in images of Hoechst 33258 DNA staining. Corresponding images of antibody labelling were subsequently overlaid using the ROI manager. To derive labelling indices, nuclear intensity in a minimum of 1000 cells was calculated and a threshold for labelling intensity was established on an appropriate negative control sample. Analysis of Rad51 nuclear speckles was performed using the Speckle Inspector tool from the BioVoxxel Toolbox (v2.5.1) in a minimum of 1,000 cells. Nuclei with three speckles or fewer were disregarded.

### Proximity ligation assays (PLA)

NHU cell cultures on glass 12-well slides were fixed in 10% neutral buffered formalin for 10 min before permeabilisation in PBS containing 0.5% (w/v) Triton X-100 for 30 min. PLA were performed using the Duolink® In Situ Red kit as per the manufacturer’s instructions (Sigma).

### Protein lysate collection

Cell cultures were scrape-harvested in lysis buffer containing 0.2% (v/v) protease inhibitors (Protease Inhibitor Cocktail set III, Calbiochem). Lysis buffer comprised: 25 mM HEPES-KOH (pH 7.5), 10% glycerol, 150 mM NaCl, 0.5% Triton X-100, 1 mM ethylenediaminetetraacetic acid. Lysates were sonicated and centrifuged at 13,000 g. A bicinchoninic acid (BCA) protein assay (ThermoScientific) was used to normalise loading into western blots and deaminase assays.

### Western blotting

Fifty µg of protein lysate per lane was resolved on NuPAGE gels using the Novex electrophoresis system (Invitrogen) at 200 V. Electrotransfer to PVDF-FL membranes (Millipore) was completed in a Tris–glycine buffer at 20 V for 2 h at 4 °C before appropriate blocking. Primary antibody details are provided in Supplementary Methods. Membranes were labelled with the appropriate IRDye conjugated secondary antibody (LI-COR) and visualised by epifluorescent infrared illumination at 700 and/or 800 nm using the Odyssey Sa scanner and software (LI-COR). Densitometry was performed using Image Studio Lite Ver 5.0 software (LI-COR). Cropped western blots from a single representative donor are shown in the main figures with full blots from all donors provided as supplementary figures. Western blots were loaded with equal protein amount in every lane and correct loading/transfer was confirmed by probing for β–actin (Supplementary Fig. 2).

### Deaminase Activity Assays

Protein lysate [1 μg/μL] was RNase A digested at 37 °C for 15 min following addition of 1 μg RNase A (Qiagen) per 25 μg protein. In Supplementary Fig. 24, the RNase digestion was omitted and 100 ng/μL of urothelial RNA was added to achieve greater selectivity for APOBEC3A (as previously described [31]). 10 μg protein lysate was mixed with 1 pmol ssDNA substrate (details in Supplementary Methods; IDT, Germany) and 0.75 U uracil-DNA glycosylase (UDG; New England Biolabs) in a total volume of 12 μL and incubated at 37 °C for 1 h. 10 μl 1 M NaOH was added and samples incubated for 15 min at 37 °C. Finally, 10 μl 1 M HCl was added to neutralize the reaction and samples were separated by electrophoresis through 15% urea-polyacrylamide gel electrophoresis gels in Tris-borate-EDTA (1x) at 150 V for 2 h. Gels were visualised by epifluorescent infrared illumination at 700 nm using the Odyssey Sa scanner and software (LI-COR). Densitometry was performed using Image Studio Lite Ver 5.0 software (LI-COR).

### Apurinic/apyrimidinic sites assay

Genomic DNA was extracted from 2–3 independent cultures of NHU cells from each of 5 independent donors using Nucleospin Tissue (Machery-Nagel) spin columns. Apurinic/apyrimidinic (AP) sites were quantified using the Oxiselect DNA damage ELISA kit (AP sites; STA-324; Cell Biolabs Inc. San Diego, CA, USA), according to manufacturer’s instructions. A standard curve of aldehyde reactive probe DNA was used to quantify the number of genomic AP sites. The assay was performed using T47D breast cancer cell DNA as a positive control for AP sites, as we have previously described [50].

### Statistical analysis

Data were assessed for statistical significance using paired tests which account for the matched genetic backgrounds of donor cell lines (mRNAseq data was assessed in Sleuth v0.30.0 [48] and non-mRNAseq data was analysed in Graphpad Prism v8.3.0 software). In paired testing, any sample missing one side of the comparison is excluded, such as in donors 6 and 8 where the interferon arm of the study was not performed. The cell culture destined to deliver the control protein lysate for Donor 4 was lost to a bacterial infection and therefore not included in “control vs BKPyV” statistical analysis but the other conditions are shown for reference. On all graphs statistical *p* or *q* value significance is represented as follows: * < 0.05, ** < 0.01, & *** < 0.001.

## Supplementary information

Supplementary Methods and Figures

Supplementary Table 1

## Data Availability

All mRNAseq data that support the findings of this study have been deposited in the NCBI GEO database with the accession code GSE174244. No unique code was used in this manuscript and all code employed is freely available from the cited sources.
